# Supplementary material for: Deciphering response dynamics and treatment resistance from circulating tumor DNA after CAR T-cells in multiple myeloma
Source: Nat Commun. 2025 Feb 20;16:1824. doi: 10.1038/s41467-025-56486-6 (PMC11842827; doi:10.1038/s41467-025-56486-6)
Supplement: Supplementary file 1 — Supplementary Information [file 41467_2025_56486_MOESM1_ESM.pdf]

Supplementary Materials

Supplementary Methods

Patients; cont.

Newly diagnosed patients were enrolled prior to receiving chemotherapy and only pre-treatment patient data and samples were considered for these patients. Relapsed/refractory patients were enrolled prior to start of a next line of therapy. Patients undergoing CAR T-cell therapy were enrolled prior to CAR T-cell infusion. High-risk cytogenetics were defined as positive FISH test in t(4;14), t(14;16), t(14;20), del(17p) or gain(1q). Oligo/non-secretory disease was defined as cases with M-spike less than 0.5 g/dL and involved free light chain less than 10 mg/dL. All patients were followed longitudinally following therapy and samples were collected sequentially prior to, during, and after therapy when available. For BCMA-CAR patients, time to progression (TTP) was defined as the length of time from CAR T-cell infusion until the disease progression. A patient with an early progression, or “early progressor” was defined as a subject who experienced disease progression within 90 days of CAR T-cell infusion. Median follow-up for the BCMA-CAR cohort was calculated based on TTP using the reverse Kaplan-Meier method.

Sample Collection and Processing; cont.

Bone marrow aspirate samples were collected in K_2EDTA tubes (BD, Franklin Lakes, NJ) and processed within 24 hours. Bone marrow mononuclear cells (BMMCs) were isolated using Ficoll-Paque (Cytiva, Marlborough, MA) according to the manufacturer’s protocol. CD138-positive cells were isolated using MACS Cell Separation using anti-CD138 microbeads (Miltenyi, Gaithersburg, MD). 20 uL of anti-CD138 microbeads were used per total of $2x10^7$ cells with 1:5 dilution by liquid volume. BMMC and CD138-positive cells were cryopreserved in freezing media (90% FBS, 10% DMSO) or CryoStor (Stemcell Technologies, Vancouver, BC) and stored in liquid nitrogen. Cell-free DNA (cfDNA) was extracted from 1.5-8 mL of plasma using the QIAamp Circulating Nucleic Acid Kit (Qiagen, Hilden, Germany), according to the manufacturer’s protocol. Cellular DNA from either plasma depleted whole blood, BMMC, or bone marrow CD138-positive cells was isolated using the QiAmp DNA Micro Kit (Qiagen) according to the manufacturer’s protocol. Following isolation, cellular DNA was mechanically fragmented to a target size of 170-bp using Covaris S2 sonicator, or enzymatically fragmented using KAPA HyperPlus Kit (Roche, Pleasanton, CA), according to the manufacturer’s protocol. DNA was quantified using the Qubit dsDNA High Sensitivity Kit (Thermo Fisher Scientific, Waltham, MA) and fragment length was assessed using a Bioanalyzer System (Agilent, Santa Clara, CA).

Identification of frequently mutated genomic regions in MM

WGS data were obtained as follows. For DLBCL, data was downloaded from the International Cancer Genome Consortium (ICGC) data portal on May 7th, 2018. For MM, data were downloaded from dbGAP (accession phs000348.v2.p1)[1] on Feb 12th, 2021. All samples had somatic mutations called using matched tumor and normal samples. In order to identify frequently mutated regions, we identified SNVs within 1-kb regions and

fraction of patients having SNVs in each binned area was calculated. Regions with more than 2 percent of cases harboring SNVs in either MM or DLBCL are depicted in Fig. 2A and Fig. S1, respectively.

Evaluating limit of detection by MM CAPP-Seq

The limit of detection at which a sample would be 95 percent likely to be detected (LOD95) using MM CAPP-Seq panel was calculated based on the number of SNVs detected from each genotyping sample. We calculated LOD95 using only coding genes in the panel versus including non-coding genomic regions (i.e., all variants).

The LOD95 was calculated as follows. First, the error-rate of tracking variants was estimated as 4×10^{-5} based on prior reports.[2] Using this background error-rate, we then assessed for each sample what the 95th percentile of the expected number of detected reads would be in a negative sample from the binomial distribution, using the MATLAB function 'binoinv'. Here, we assumed 2500x sequencing depth, and used the number of tumor variants assessable considering either coding variants alone, or all variants. This yielded the threshold number of detected reads above which tumor signal can be detected with >95% specificity.

Then, we determined the minimum tumor fraction above which we would expect to see this number of detected reads or more, given the sequencing depth above. We performed this using the right-hand portion of the binomial cumulative distribution function 'binocdf' in MATLAB, with minimization using the 'fmincon' package. This procedure was performed for each patient's specific number of coding variants, versus all variants, and is shown in Fig 2E.

Library Preparation and Sequencing

Barcoded DNA sequencing libraries were prepared as described in detail previously,[3] followed by hybrid capture. Samples from patients at new diagnosis or relapse of myeloma and subsequent samples during treatment were captured using custom panels as described in the main text. Libraries were sequenced on an Illumina HiSeq or NovaSeq platforms with 2x150bp paired-end reads.

Sequencing Data Analysis and Variant Calling

FASTQs files were demultiplexed based on 8-bp dual sample barcodes and reads were mapped to a customized version of the human reference genome (hg19) augmented with the lentiviral sequence for ide-cel and cilta-cel using Burrows-Wheeler Alignment (BWA) ALN. Molecules were deduplicated with error-suppression using a custom pipeline as previously described.[2,3] The median sequencing depth after deduplication for cfDNA, germline, and BMMC samples were 2507x, 2649x, and 3640x, respectively. Following this, somatic single nucleotide variants (SNVs) were called using a custom variant algorithm optimized for cfDNA as previously described.[2] Briefly, the sequencing data undergoes vigorous filtering including 1) matched normal to account for germline mutations, 2) a panel of healthy controls to account for stereotyped artifactual mutations, 3) molecular barcoding and in silico error suppression to account for sequencing. For germline filtering, we used PBMC as germline and somatic variants

were considered i) if it is only present in cfDNA with allele frequency greater than 0.3% and not in germline, or ii) if allele frequency is greater than 0.3% and at least 5 times higher in the cfDNA compared to germline. The latter SNVs were considered to be tumor-derived given that circulating tumor cells can be detected in MM. For patients with plasma cell leukemia, who had circulating plasma cells by manual differential at the time of sample collection (MM-31, MM-33), germline-free SNV calling was employed as PBMC was contaminated by plasma cells. Additional filtering considered read support in a panel of healthy controls as previously described.[4] Missense, frame-shift, nonsense 5' or 3'UTR, and splice-site variants were considered "non-silent."

Evaluating false-positive rate of de novo genotyping and tumor-informed ctDNA tracking
False-positive rate (FPR) and specificity were assessed as following. As our methods perform two distinct analysis: i) de novo genotyping of pre-treatment samples without knowledge of tumor-derived SNVs, followed by ii) assessment of residual ctDNA with the knowledge of tumor-derived SNVs, we assessed FPR and specificity in both steps.
(i) De novo genotyping:
For *de novo* genotyping, we assessed FPR and specificity by examining performance of our assay in samples without significant measurable myeloma, using the same genotyping rules we applied to patient samples in our cohort.
Furthermore, we evaluated the specificity of our assay at a base-pair level resolution given the size of our panel.
ii) Measuring and tracking ctDNA levels
FPR of tracking ctDNA was evaluated in a training cohort of healthy controls. Monte-Carlo testing was performed using the detected SNVs from each genotypable patient in the study cohort against controls and false-positive rate was calculated as:

$$false\ positive\ rate = \frac{\text{Number of significantly postive tests}}{\text{Number of tests}}$$

A FPR of 5% was set by adjusting p-value threshold for detection.
We then validated the FPR using 4 additional withheld healthy control plasmas by performing Monte-Carlo testing.

Genotyping and ctDNA tracking
Genotyping of each patient's myeloma was performed using bone marrow or plasma samples using pre-treatment samples or at relapse. For 2 patients in CAR cohort, whose pre-CAR samples were unavailable, samples at the earliest timepoints, day 2 and day 7, respectively, were used for genotyping. For 3 patients, a relapse plasma sample was used for genotyping as pre-CAR samples had SNVs<15. ctDNA was then tracked in serial samples for each patient throughout the course of therapy. For CAR cohort, we attempted to collect samples upon evaluation, pre-lymphodepletion chemotherapy, day 0, 7, 14, 21, 28, 60, 90, and upon relapse.

ctDNA Quantification and Monitoring

ctDNA levels were measured in haploid genome equivalents per milliliter (hGE/mL), calculated as the product of total cell-free DNA concentration and the mean allele-fraction of somatic mutations. ctDNA levels were monitored in individual samples using a previously described Monte-Carlo-based ctDNA detection index.[2]

Copy Number and Structural Alterations Analysis

Genome-wide somatic copy number alterations (CNAs) were called using both on-target (~60-80% of reads) and off-target (~20-40%) sequencing reads as previously described (Copy number ANomaly Assessment and RecoverY; CANARy).[3,5] CNAs specific to individual genes and at the level of specific chromosome arms were considered for further analysis. Z-scores were normalized against a panel of cell-free DNA of healthy controls (n=26) and diploid germline DNA (n=64). Significant copy number gain or loss was considered when false-discovery rate (q-score) was less than 0.1. CNAs were confirmed by WGS data where available. CNAs detected by CANARy were compared with clinical fluorescence *in situ* hybridization (FISH) data. FISH data was collected from the same day of research sample collection, or from the latest date available if it was not performed on the same day. Among our cohort, there were 63 patients who had FISH data, with 30 patients having same-day FISH, and 33 patients having historical data.

Mutational Signatures of detected SNVs

To assess the mutational processes associated with SNVs detected from genotyping samples, mutational signatures of single base substitutions (SBSs) were enumerated using the R package 'deconstructSigs'.[6] Frequently detected intronic nonsynonymous SNVs (*i.e.* in *BCL7A, BCL6, IGLL5, LPP, IGH, IGL, IGK*) as well as frequently detected nonsynonymous SNVs in driver genes (i.e. *KRAS, NRAS, TP53*) were applied 'deconstructSigs' using 79 SBS signatures described in COSMIC[7] to assess the contribution of each SBS signature.

Quantification of CAR cfDNA

120 base-pair hybrid capture oligonucleotides (IDT, Coralville, IA) were designed to tile across the ide-cel and cilta-cel lentiviral vector sequences and used in library captures. Full sequence of ide-cel were available, while only limited regions of cilta-cel were available. The region of the vector sequence evaluable for determining depth was defined by first considering only the region between the long terminal repeats (LTR), which is the region expected to integrate into host genome. The evaluable region was then refined by comparing the depth in this region between patients known to have received CAR therapy with healthy controls and removing regions with significant depth in controls. Reads aligned to remaining evaluable regions with mapping quality > 40 were considered for further analysis. Mean depth across these regions was then calculated per sample, and CAR level was defined as CAR genomes per milliliter, which was determined as follows: (ide-cel or cilta-cel depth / human depth) * (1 hGE/3e-12 g) * (1e9 ng/1g) * plasma cfDNA concentration.

Clinical MRD assay

MRD in routine clinical practice was performed by NGS platform using ClonoSeq assay (Adaptive Biotechnologies, Seattle, WA) or multiparametric flow cytometry (Mayo Clinic, MN). Identification of dominant clones was attempted by ClonoSeq using fresh and/or archival bone marrow samples. For those specimens that were unsuccessful in identifying dominant clones, post-treatment samples were sent for multiparametric flow cytometry assay for MRD evaluation. Flow cytometry at Mayo Clinic was performed using the following antibodies: Tube 1: CD138, CD27, CD38, CD56, CD45, CD19, CD117, and CD81. Tube 2: CD138, CD27, CD38, CD56, CD45, CD19, cyKappa, and cyLambda. Abnormal plasma cell populations are detected through demonstrating CD38 (multiepitope) and CD138 positivity along with immunoglobulin light chain restriction (ie, the presence of either predominately kappa or predominately lambda light chains) and abnormality of CD56, CD117, CD27, CD81, CD19 and/or CD45 expression.[8]

Flow cytometry for quantification of CAR T-cells

In patients who underwent CAR T-cell therapy, a flow cytometry assay incorporating a PE fluorochrome labeled human BCMA/TNFRSF17 protein was used to detect BCMA-specific CAR T-cells sequentially after CAR T-cell infusion.

Histology and Immunohistochemistry

Bone marrow core biopsy samples were fixed in Bouin's solution, decalcified, embedded in paraffin and sectioned. Immunohistochemistry was performed on four-micron sections using standard automated methods including instrument-based deparaffinization, peroxidase blocking, antigen retrieval, primary and secondary antibody incubation, detection and counterstaining. Assays were performed on Roche Ventana (Tucson, AZ) Ultra instruments using Ventana Optiview detection, or Leica (Buffalo Grove, IL) Bond III instruments using Leica Polymer Refine detection. Antibodies CD138 clone B-A38 from Ventana/Cell Marque (catalog 760-4248) and BCMA clone D-6 from Santa Cruz Biotech (catalog sc-390147) where used.

Emergent Copy Number Alteration Analysis

To enable identification of emergent or clonally selected CNAs, we assessed cases of patients receiving CAR T-cell therapy with paired samples at both the pretreatment and relapse time-points. CNAs were identified in the paired samples with each alteration assigned a Z-score representing the magnitude of the alteration as described above. We then calculated a distribution across all identified CNAs as:

$$T_i = Z_{AMP_{i,relapse}} - Z_{AMP_{i,pretreatment}}$$

with a corresponding distribution for amplifications and deletions. We then calculated a Z-score of these T_i differences:

$$Z_i = \frac{T_i - \mu_T}{\sigma_T}$$

to determine the magnitude of clonal selection for each copy number variant. This Z-score (Z_i) represents the degree of selection for a given variant at relapse relative to all other variants.

We then tested the degree to which recurrent CNAs were selected across the entire population in our study. To perform a Wilcoxon rank-sum test for a given locus *k*, for a set of variants across *n* subjects, we identified the set of Z values $\{Z_{k,1}, Z_{k,2} \ldots Z_{k,n}\}$. This set of Z-scores was then compared to all other alterations using a Wilcoxon rank-sum test.

Analysis of prognostic significance of pre-treatment genomic alterations on CAR-T therapy

To assess the significance of pre-treatment genomic alterations on clinical outcomes after CAR-T therapy, we considered pre-treatment SNVs and CNAs with false-discovery q-score of less than 0.05 that are present in our CAR-T cohort. We then assessed the impact of these genomic alterations on TTP using Cox proportional hazards modeling.

Supplementary Note

False-positive rate of de novo genotyping and tumor-informed ctDNA tracking

We evaluated false-positive rate (FPR) and specificity of our assay (Supplemental Methods). We assessed FPR and specificity of our analysis of i) *de novo* genotyping of pre-treatment samples without knowledge of tumor-derived SNVs, and ii) assessment of residual ctDNA with the knowledge of tumor-derived SNVs. For i), we genotyped samples in remission (n=23) using the same genotyping rules we applied to patient samples in our cohort. In contrast to samples at the time of active myeloma (n=54) with median 86 detected SNVs (median AF 2.1%), samples in remission detected median of 0 SNVs (median AF 0.74%) (Fig. S2). The specificity was evaluated at a base-pair level resolution. Among 23 samples in remission, there were only 13 total SNVs identified, representing a specificity of >99.99% across 560 kilo-bases. For ii), FPR of tracking ctDNA was evaluated in a training cohort of healthy controls (n=26) against SNVs detected from each genotypable patient (n=55; 26 x 55 tests). A p-value threshold for significant ctDNA detection was then set to keep an FPR of 5%. We then used a validation cohort (n=4, 4x55 tests), and observed the FPR was 5.6%, further validating 95% specificity of the assay.

Supplementary Tables

Table S1 Genes included in MM CAPP-Seq panel

ACTG1	*CD274*	*FBXW7*	*IRF4*	*NFKBIA*	*RIPK1*
AKT1	*CD38*	*FCRL5(FcRH5)*	*JAK2*	*NR3C1*	*SETD2*
ARID2	*CDKN1B*	*FGFR3*	*KDM6A*	*NRAS*	*SF3B1*
ATM	*CDKN2A*	*FSIP2*	*KLHL6*	*PDCD1*	*SLAMF7*
B2M	*CDKN2C*	*GPRC5D*	*KMT2D*	*PIK3CA*	*SP140*
BCL2	*CKS1B*	*HIST1H1B*	*KRAS*	*PIM1*	*STAT3*
TNFRSF17 (BCMA)	*CRBN*	*HIST1H1C*	*LTB*	*PPM1D*	*TP53*
BIRC2	*CUL4B*	*HIST1H1E*	*MAF*	*PRDM1*	*TRAF2*
BIRC3	*CXCR4*	*IDH1*	*MAFB*	*PRKD2*	*TRAF3*
BRAF	*CYLD*	*IDH2*	*MAX*	*PSMB5*	*WHSC1*
BTG1	*DIS3*	*IGLL5*	*MYC*	*PSMD1*	*XBP1*
CCND1	*DUSP2*	*IKBKB*	*MYD88*	*PTEN*	*XPO1*
CCND3	*EGR1*	*IKZF1*	*NF1*	*PTPN11*	
SCD1(CD138)	*FAM46C*	*IKZF3*	*NFKB2*	*RB1*	

Table S2 SNVs covered by clinical Heme-STAMP and CAPP-Seq panels that were reported by Heme-STAMP

	Gene	Chr	Position	VAF (%)	Detected by CAPP-Seq
MM-07	ATM	11	108201117	37	1
MM-11	DNMT3A	2	25464578	10	1
MM-24	PRDM1	6	106553257	48	1
MM-25	KRAS	12	25380275	1	0
MM-27	CCND1	11	69456230	10	1
MM-27	MYC	8	128752641	6	1
MM-30	EGR1	5	137801708	12	1
MM-30	EGR1	5	137801642	10	1
MM-31	NRAS	1	115256529	10	1
MM-31	PPM1D	17	58740530	49	1
MM-31	CCND1	11	69456211	15	1
MM-31	IRF4	6	394972	7	1

Table S3 Treatment regiments of the cohort who have ctDNA data on pre-treatment and D28 (corresponding to Figure 4F)

ID	Current line of therapy	Treatment
**MM-05**	6	Venetoclax/carfilzomib/dexamethasone
**MM-06**	6	Cyclophosphamide/etoposide/cisplatin/dexamethasone
**MM-07**	1	Daratumumab/dexamethasone
**MM-11**	17	Selinexor/dexamethasone
**MM-15**	1	Daratumumab/bortezomib/dexamethasone
**MM-19**	2	Daratumumab/carfilzomib
**MM-20**	1	Carfilzomib/lenalidomide/dexamethasone
**MM-24**	1	Daratumumab/cyclophosphamide/bortezomib/dexamethasone
**MM-28**	1	Daratumumab/bortezomib/lenalidomide/dexamethasone
**MM-32**	3	Cyclophosphamide/doxorubicin/cisplatin/etoposide
**CAR-01**	10	Ide-cel
**CAR-02**	6	Ide-cel
**CAR-03**	6	Ide-cel
**CAR-04**	7	Ide-cel
**CAR-05**	16	Ide-cel
**CAR-06**	7	Ide-cel
**CAR-07**	5	Ide-cel
**CAR-08**	5	Cilta-cel
**CAR-10**	5	Ide-cel
**CAR-11**	10	Ide-cel
**CAR-12**	9	Ide-cel
**CAR-14**	8	Ide-cel
**CAR-15**	9	Ide-cel
**CAR-17**	12	Ide-cel
**CAR-18**	6	Cilta-cel
**CAR-19**	6	Cilta-cel
**CAR-20**	16	Cilta-cel
**CAR-21**	5	Cilta-cel
**CAR-22**	9	Ide-cel
**CAR-24**	11	Ide-cel
**CAR-33**	10	Ide-cel

Table S4 Demographics of patients undergoing CAR T-cell therapy. Ide-cel, idecabtagene vicleucel; cilta-cel, ciltacabtagene autoleucel.

	Median (range) or N (%)
Age (yr)	65 (30-81)
Sex, female	14 (39%)
Type of therapy	
- Ide-cel	30 (83%)
- Cilta-cel	6 (17%)
R-ISS, I	10 (28%)
II/III	25 (69%)
unknown	1 (3%)
High-risk cytogenetics	18 (50%)
Prior lines of therapy (median)	6 (4-15)
History of extramedullary disease	21 (58%)
Oligo/non-secretory disease	7 (19%)
Prior exposure to BCMA-targeted therapy	5 (14%)
Bone marrow involvement (%)	5 (0-90)

Table S5 BCMA expression in the bone marrow and BCMA copy number estimated by CAPP-Seq

	BCMA expression by bone marrow IHC prior to CAR-T	Copy Number estimated by CAPP-Seq	Inferred Tumor Copy Number of the dominant clone
**CAR-04**	80% positive, 20% dim - negative	0.05	0
**CAR-15**	Not evaluable	0.65	1
**CAR-10**	100% positive	1.2	1
**CAR-08**	100% positive	0.72	1
**CAR-03**	100% positive (predominantly perinuclear and dim cytoplasmic)	0.51	1

Supplementary Figures and Figure Legends

Figure S1

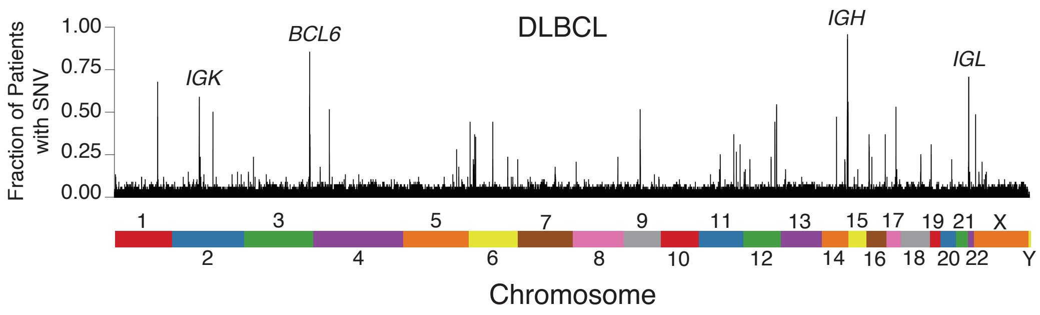

Frequently mutated genomic regions in DLBCL. A bar plot of distribution of single nucleotide variants in diffuse large B-cell lymphoma (DLBCL) from WGS data. The genome was divided into 1,000-bp bins, and the fraction of patients with a variant in each bin was calculated.

Figure S2

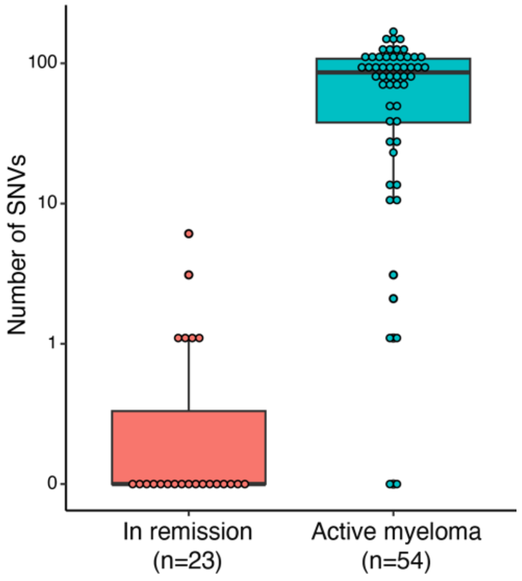

***De novo* genotyping of samples in remission vs active disease.** Box plot showing the performance of panel-wide genotyping. SNVs were identified from patients during time-points of remission (n=23), when no detectable disease should be present, and active myeloma (n=54), when mutations should be identified. See Supplemental Materials for additional details.

Figure S3

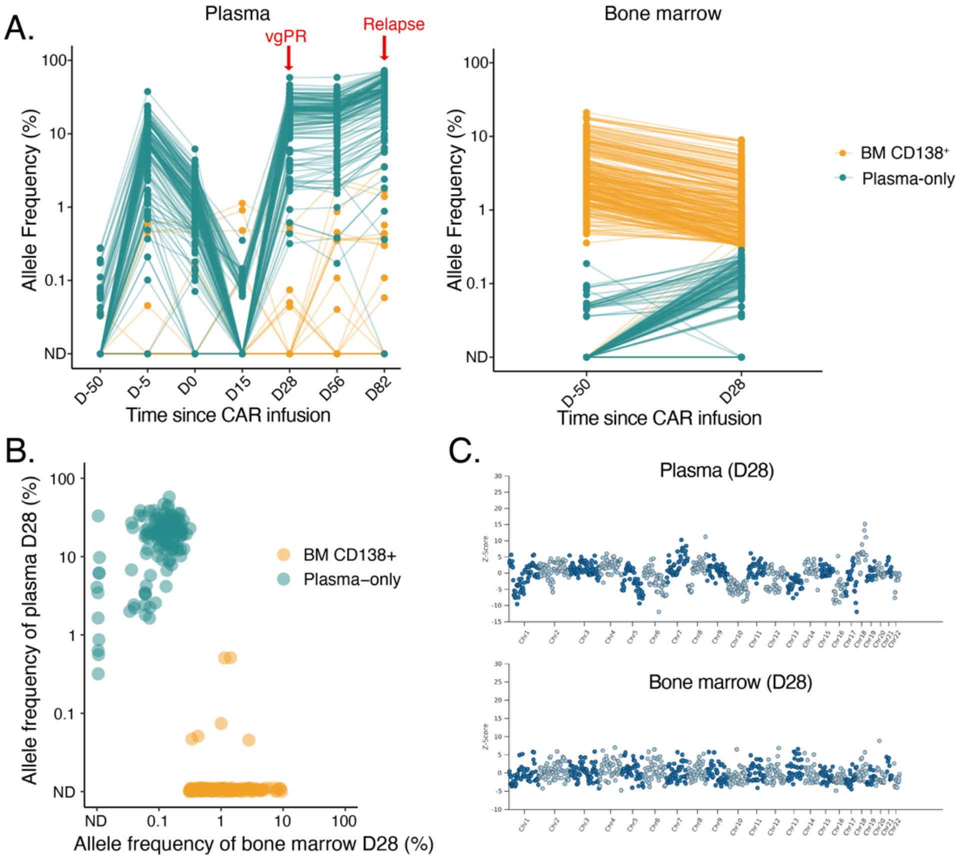

ctDNA potentially captures spatial heterogeneity of multiple myeloma. SNV dynamics of ctDNA vs bone marrow during CAR-T therapy in CAR-06, who relapsed on day 82. (A) Dynamics of each SNV in the plasma and bone marrow. Orange dots are SNVs detected in the BM at VAF>0.3%, teal dots are SNVs detected only in the plasma at VAF>0.3%. (B) A scatter plot of allele frequencies of each SNVs in the plasma vs bone marrow on day 28. (C) Copy number plot of plasma vs bone marrow on day 28 across the whole genome.

Figure S4

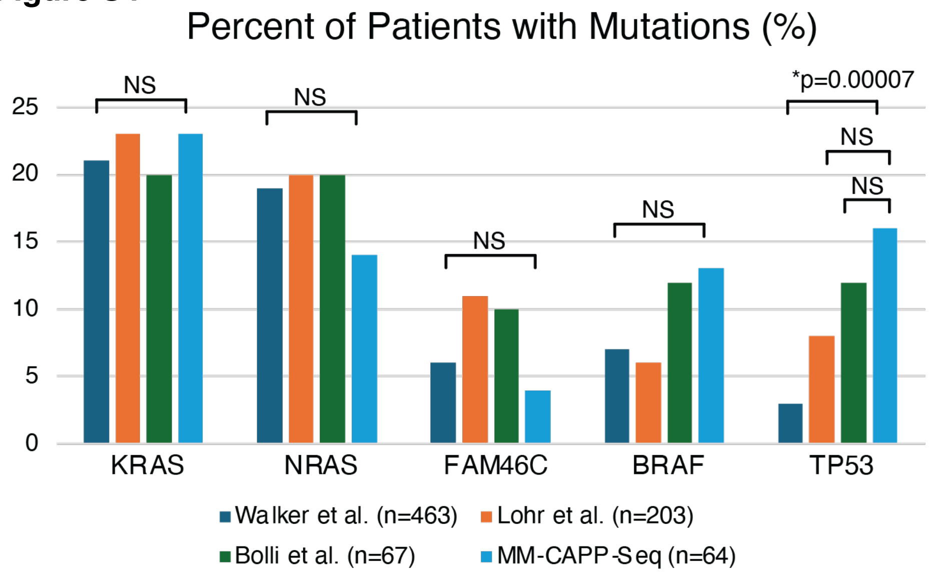

Driver mutations detected by CAPP-Seq are concordant with prior literature. Fraction of patients with main driver mutations detected by previously reported WES results[1,9,10] vs our study cohort analyzed by CAPP-Seq.

Figure S5

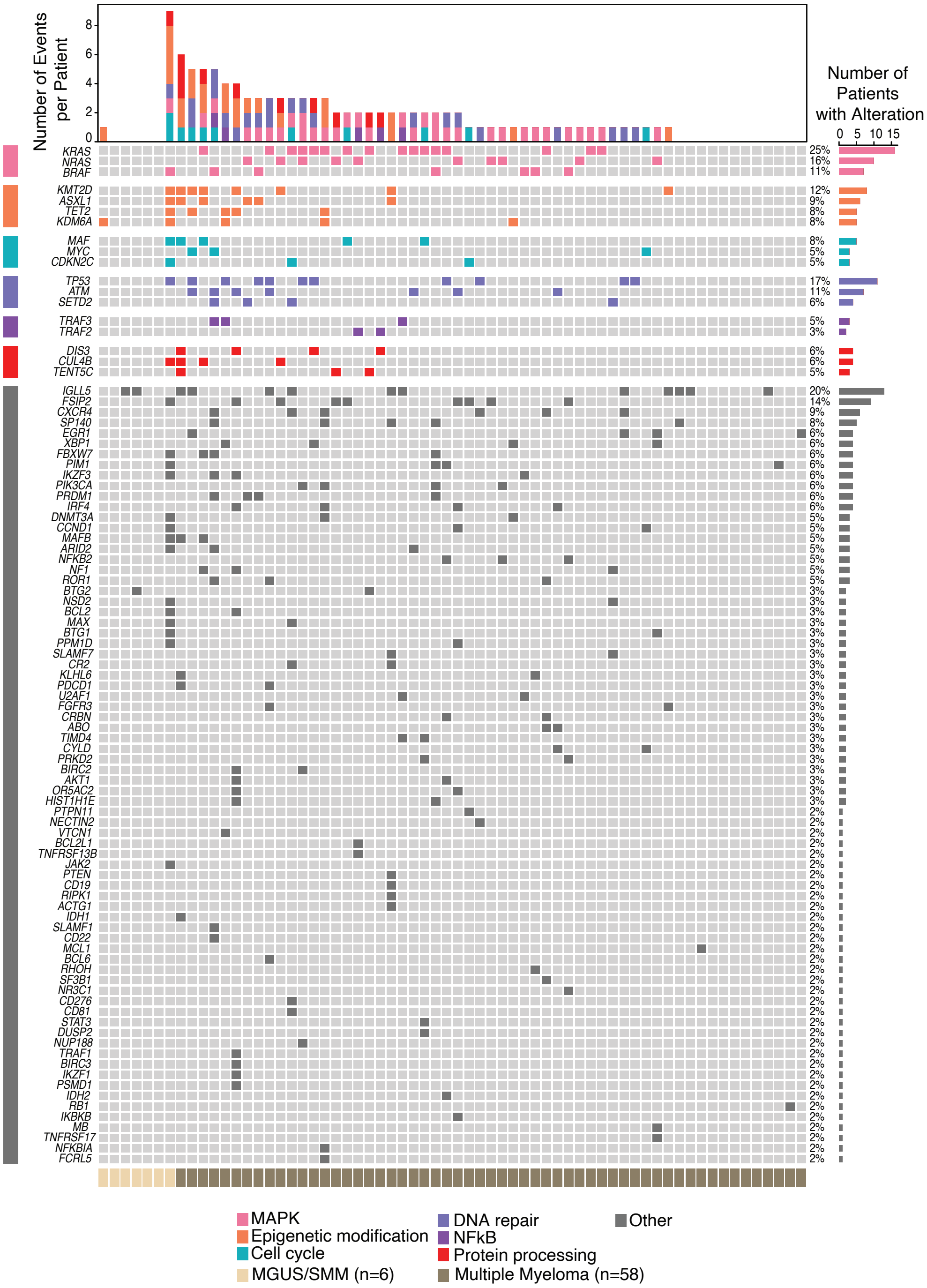

Full oncoprint showing the coding mutations detected in our cohort (n=64). All coding SNVs detected in any patient are shown. MGUS/SMM vs. MM is identified by the color bar in the bottom. MAPK, mitogen-activated protein kinase; NF-κB, nuclear factor-κB.

Figure S6

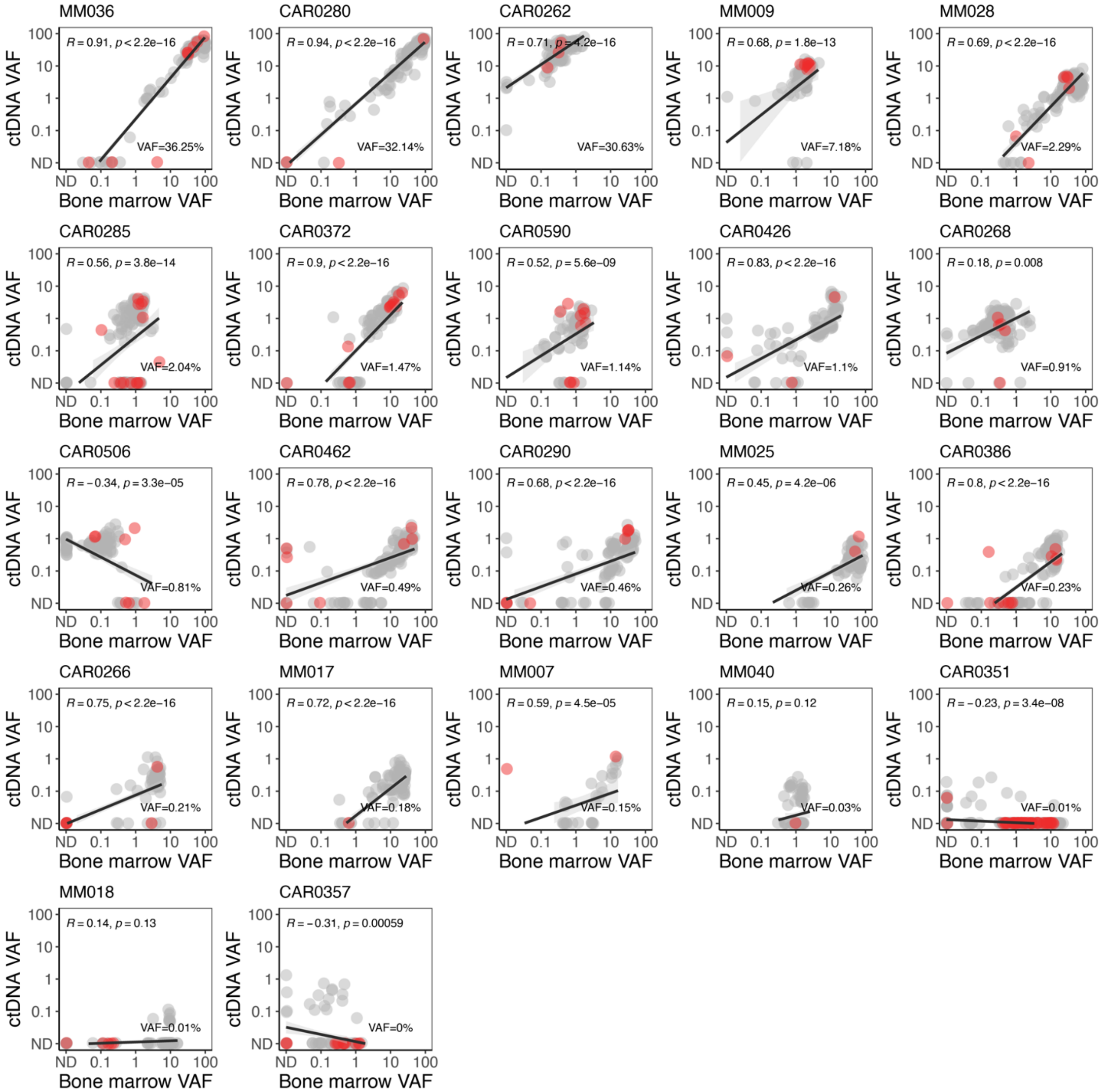

Concordance of VAF of SNVs in the bone marrow and ctDNA. Scatter plots of VAFs of coding genes (red) and non-coding regions (grey) detected from bone marrow (BM) and plasma in patients who had paired samples (n=22) ordered by mean plasma VAF. Correlation analyses were performed using Spearman's rank-order test.

Figure S7

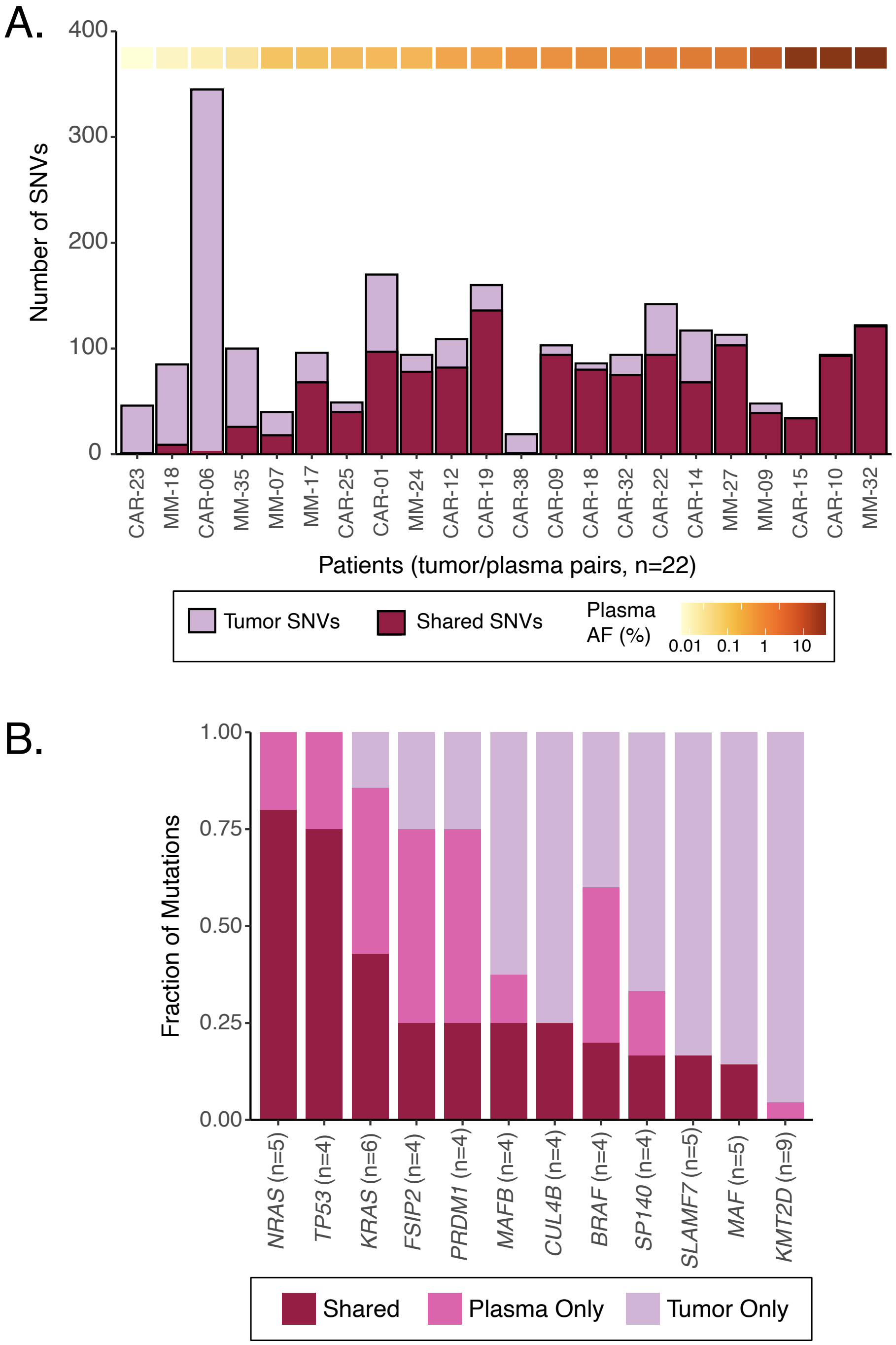

Concordance of SNVs in the bone marrow and ctDNA

A) A bar plot showing the concordance of SNVs detected in the blood plasma and paired BM. The plot shows BM SNVs per case; the bottom panel shows BM SNVs, where the bar is colored dark purple if they are also detected in the plasma. The mean AF of the plasma is shown as heat above the plot.

B) The rate of shared mutations between bone marrow and plasma are shown. Genes with mutations in at least 4 cases are shown.

Figure S8

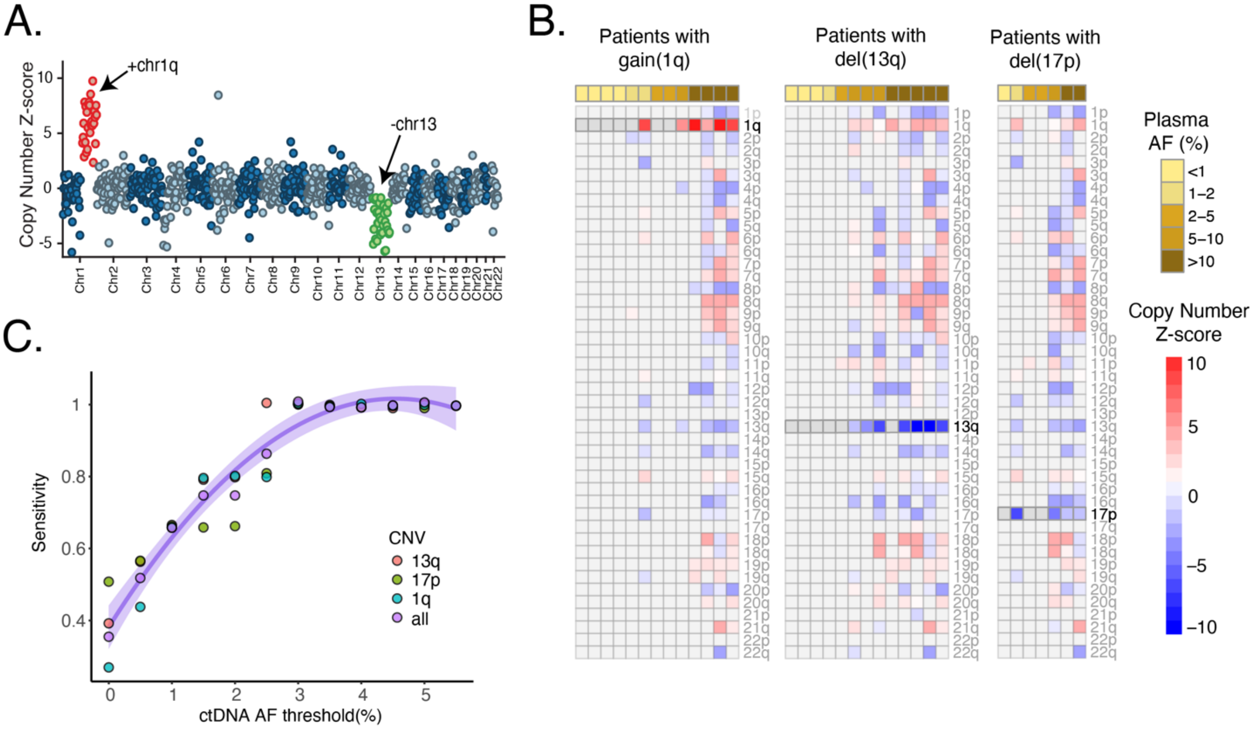

Copy number alterations detected by cfDNA

A) An example of ideogram of copy number alterations using on-target and off-target sequencing reads analyzed by CANARy.

B) Detection of gain(1q), del(13q), and del(17p) from cfDNA in patients known to have these copy number alterations by bone marrow FISH assay. Cases are ordered by AF, which is shown as heat.

C) A plot depicting the sensitivity of detection of gain(1q), del(13q), and del(17p) from cfDNA as a function of AF, with clinical FISH as a gold standard. The regression line for sensitivity of detection of all CNAs versus mean ctDNA tumor allele fraction is shown.

Figure S9

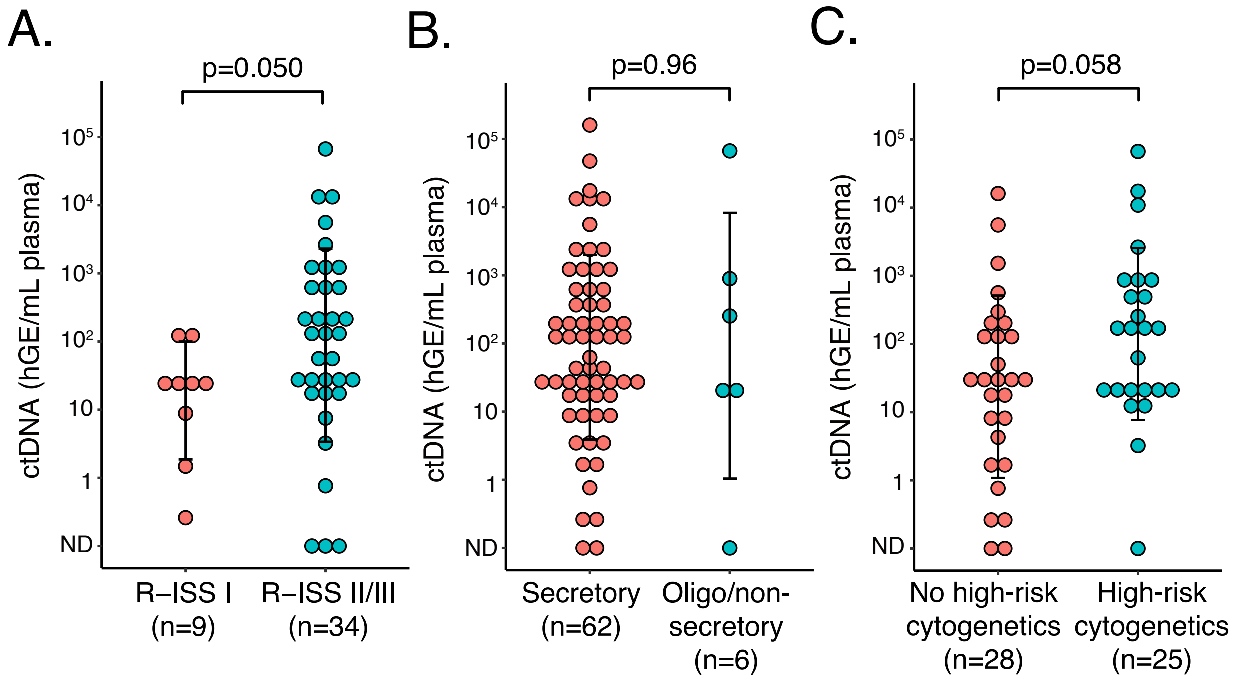

Levels of ctDNA by disease characteristics. A) Stages R-ISS I versus R-ISS II/III (p=0.050) B) Secretory versus oligo/nonsecretory (p=0.96); C) Disease with high-risk cytogenetics versus not (p=0.058). Cases with these clinical data available were included in the analysis.

Figure S10

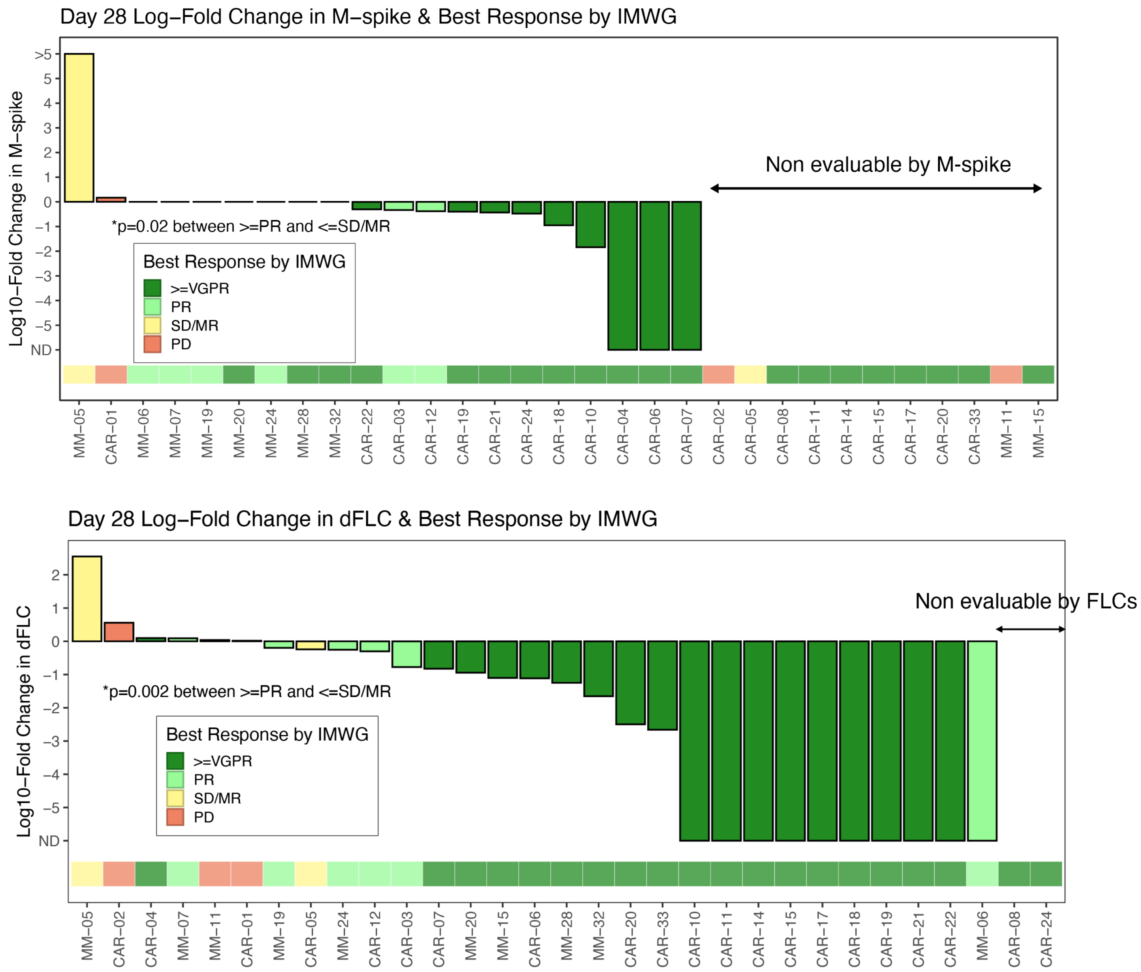

Waterfall plots demonstrating fold change of M-spike and difference between involved and uninvolved free light chains (dFLC) in association with clinical response assessed by IMWG response criteria.

Figure S11

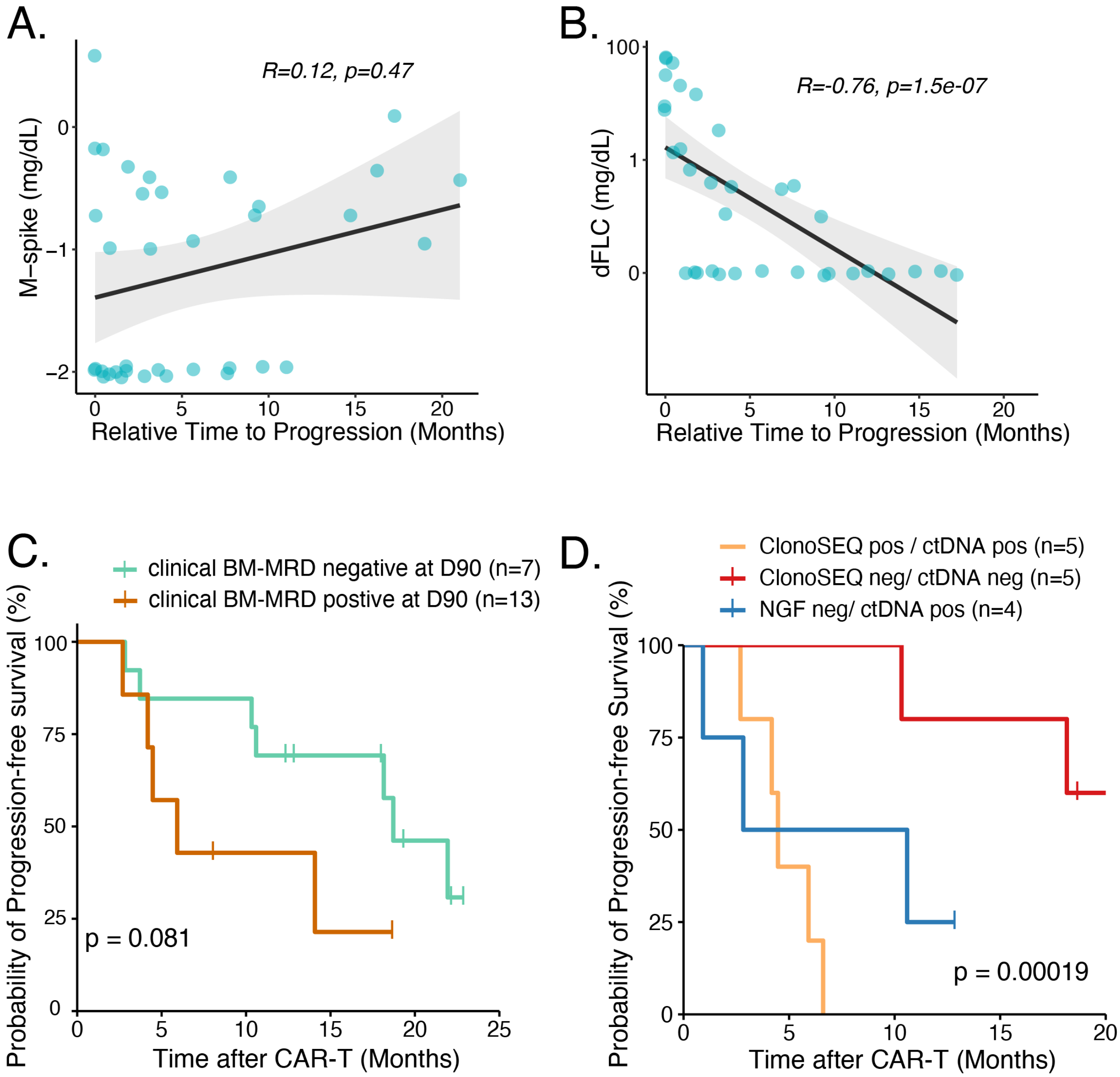

Correlation between clinical markers and clinical outcomes following CAR-T therapy. A,B) Scatter plot showing correlation between clinical markers (A. M-spike and B. difference in involved and uninvolved free light chains) post-CART and relative TTP from individual timepoints. P-value was calculated using Spearman's rank-order test. C) Kaplan-Meier analysis of PFS stratified by clinical MRD on day 90 (p=0.081). D) Kaplan-Meier analysis of PFS stratified by clinical MRD (ClonoSeq and NGF) and ctDNA.

Figure S12

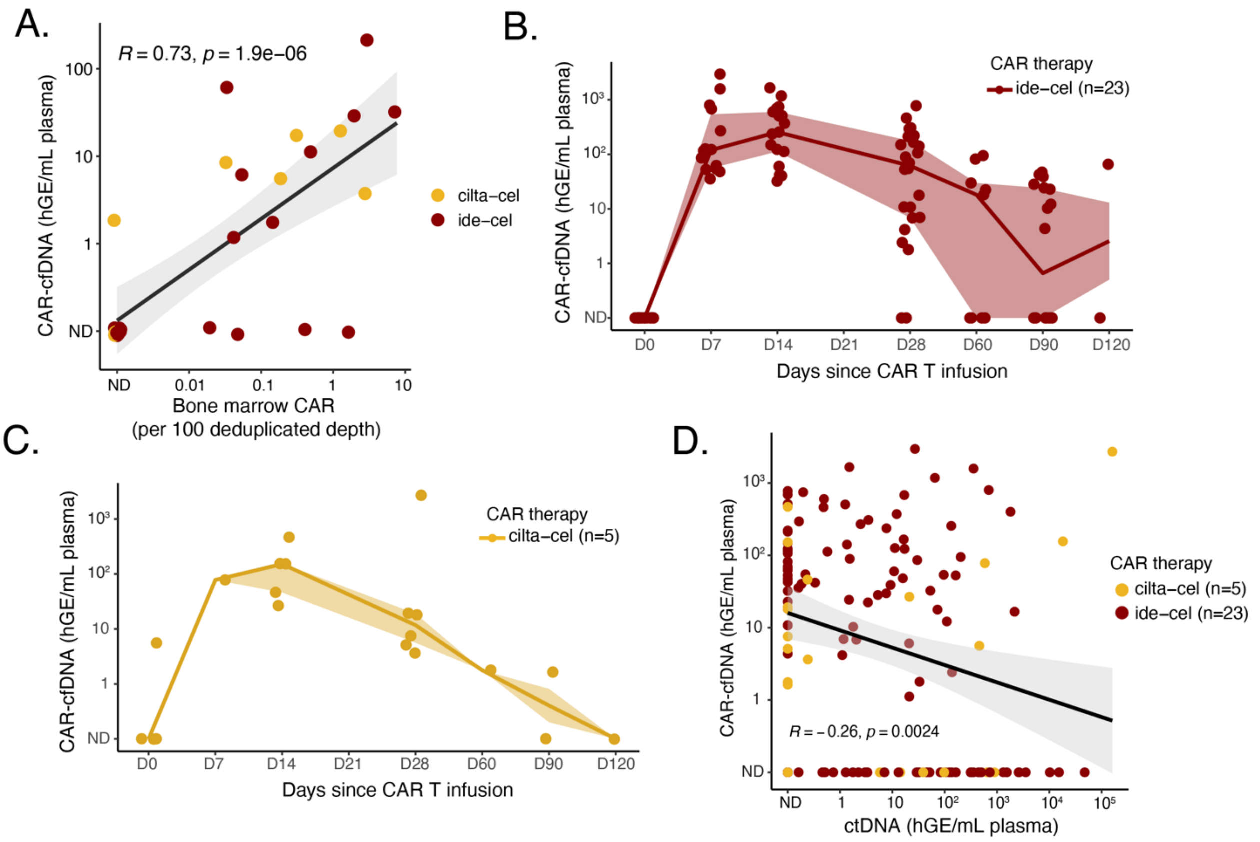

Cell-free DNA can assess CAR-T kinetics.

A) A scatter plot showing CAR-cfDNA levels and corresponding bone marrow CAR levels on patients who had paired samples post-CART therapy (*R*=0.73; p=1.9x10^{-6}). Correlation analysis was performed using Spearman's rank-order test.

B) Dynamics of CAR-cfDNA following ide-cel infusion.

C) Dynamics of CAR-cfDNA following cilta-cel infusion.

D) Correlation between CAR-cfDNA and ctDNA following CAR infusion (*R*=-0.26; p=0.0024). Correlation analysis was performed using Spearman's rank-order test.

Figure S13

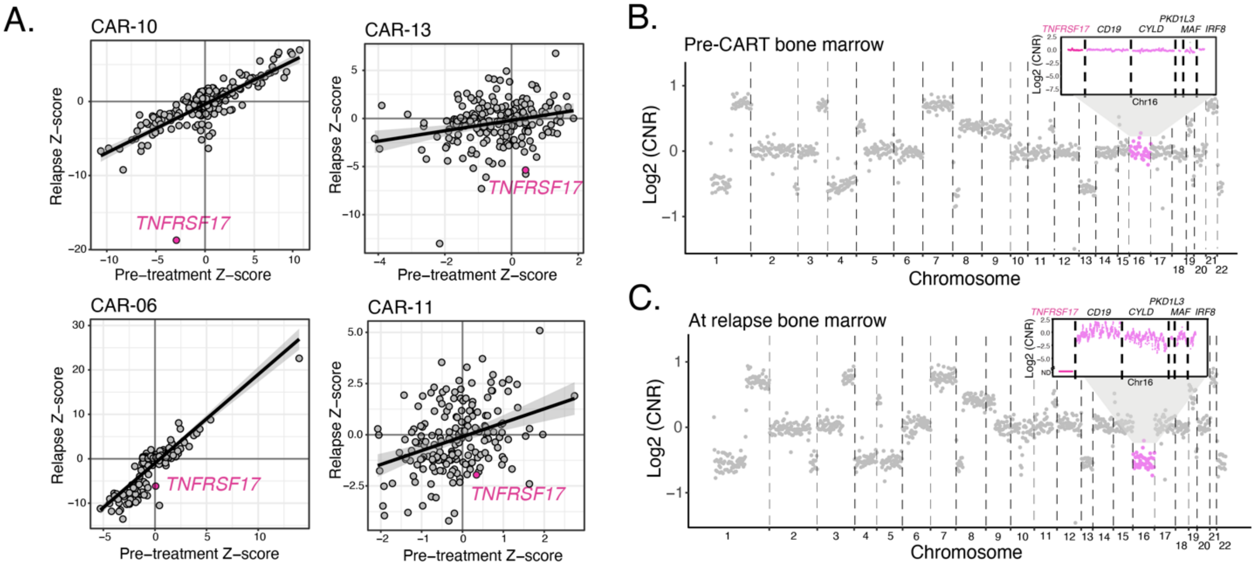

Cases of emergent *TNFRSF17*/BMCA loss following CAR-T therapy.

A) Copy number analysis at gene level comparing pre-CART and at relapse. Here, each point is a single on-target gene from our CAPP-Seq panel. Cases with emergent *TNFRSF17* loss at relapse are depicted. Copy number Z-scores at relapse (y-axis) are plotted against Z-score prior to BCMA-CAR (x-axis). Emergent loss was defined as significant FDR-corrected p-value at relapse at either the gene level or cytoband (16p13.13) level, which was not detected in the pre-treatment sample.

B, C) Genome-wide copy number profile by performing WGS on bone marrow tumor samples of CAR-10, corresponding to Fig. 6B, prior to CAR-T (B) and at relapse (C). Inserts are showing copy number profile of targeted genes obtained from CAPP-Seq, which suggests a focal *TNFRSF17* loss upon relapse. Circular binary segmentation was performed using an R package, DNACopy.

Figure S14

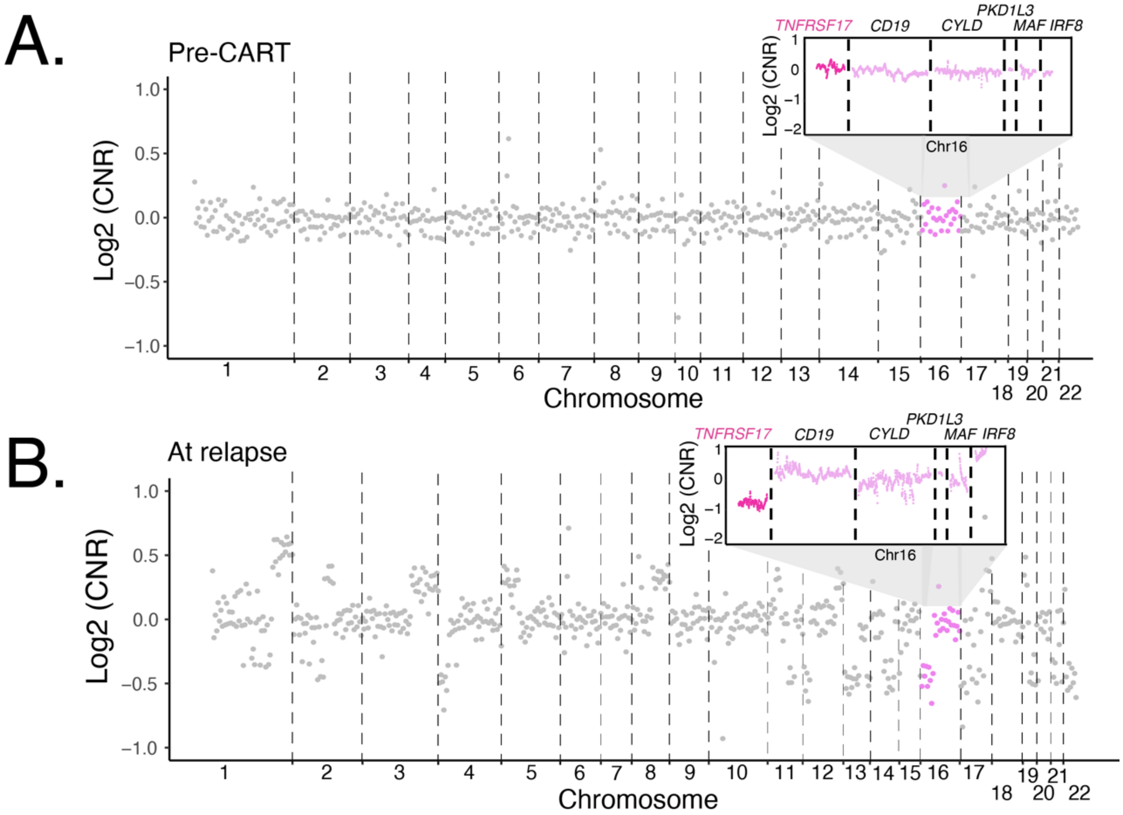

Another case of emergent focal deletion of *TNFRSF17*/BCMA detected by cfDNA following CAR-T.

Another case, CAR-13, showing an emergent focal deletion of *TNFRSF17* upon relapse. Genome-wide copy number profile of blood plasma-derived cell-free DNA after circular binary segmentation is shown from a sample prior to BCMA-CAR therapy (A) and at relapse (B). Copy number profile of targeted genes on chr16 are magnified in inserts.

Figure S15

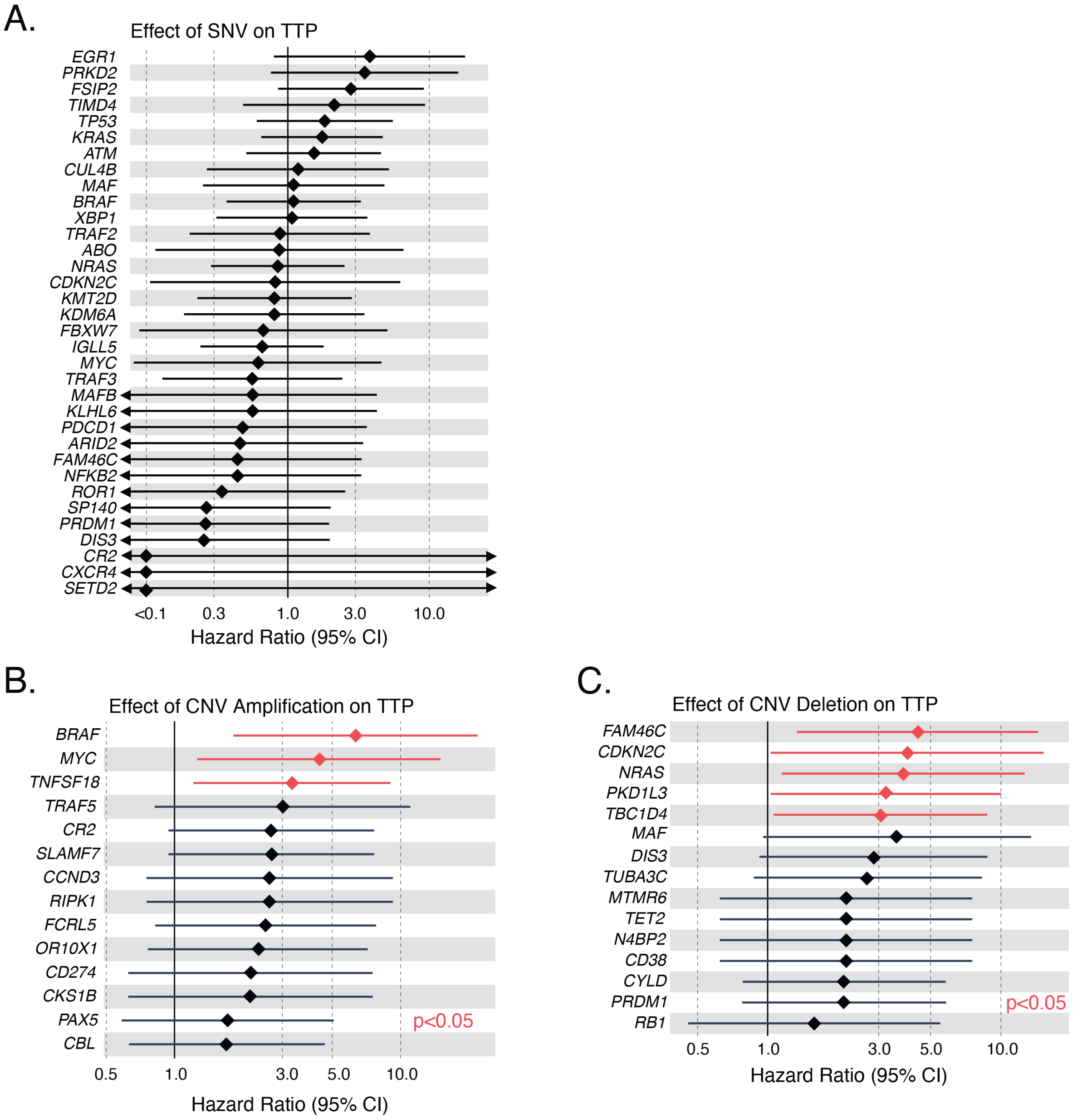

Genomic determinants of resistance following BCMA-CAR.
A) A forest plot of various single nucleotide variants affecting on time to progression. Plot depicts hazard ratios calculated by univariate Cox proportional hazards regressions model. Plot includes SNVs detected in more than one patient in the cohort. Error bars reflect 95% confidence interval. None had a statistically significant p-value of <0.05.

B, C) A forest plot of various copy number amplifications (B) or deletions (C) affecting on time to progression. Plot depicts hazard ratios calculated by univariate Cox

proportional hazards regressions model. Plot includes copy number amplifications detected in at least 4 patients in the cohort. Significant values shown in red ($p<0.05$); error bars reflect 95% confidence interval.

Supplementary References

1 Lohr, J. G. *et al.* Widespread genetic heterogeneity in multiple myeloma: implications for targeted therapy. *Cancer Cell* **25**, 91-101 (2014). https://doi.org/10.1016/j.ccr.2013.12.015

2 Newman, A. M. *et al.* Integrated digital error suppression for improved detection of circulating tumor DNA. *Nat Biotechnol* **34**, 547-555 (2016). https://doi.org/10.1038/nbt.3520

3 Chabon, J. J. *et al.* Integrating genomic features for non-invasive early lung cancer detection. *Nature* **580**, 245-251 (2020). https://doi.org/10.1038/s41586-020-2140-0

4 Kurtz, D. M. *et al.* Circulating Tumor DNA Measurements As Early Outcome Predictors in Diffuse Large B-Cell Lymphoma. *J Clin Oncol* **36**, 2845-2853 (2018). https://doi.org/10.1200/JCO.2018.78.5246

5 Palomino Mosquera, A. *et al.* Accurate Detection of Clinically Actionable Copy Number Variants in Diverse Hematological Neoplasms By Routine Targeted Sequencing: A Comparative Performance Study. *Blood* **140**, 10712-10713 (2022). https://doi.org/10.1182/blood-2022-167900

6 Rosenthal, R., McGranahan, N., Herrero, J., Taylor, B. S. & Swanton, C. DeconstructSigs: delineating mutational processes in single tumors distinguishes DNA repair deficiencies and patterns of carcinoma evolution. *Genome Biol* **17**, 31 (2016). https://doi.org/10.1186/s13059-016-0893-4

7 Alexandrov, L. B. *et al.* The repertoire of mutational signatures in human cancer. *Nature* **578**, 94-101 (2020). https://doi.org/10.1038/s41586-020-1943-3

8 Kumar, S. *et al.* International Myeloma Working Group consensus criteria for response and minimal residual disease assessment in multiple myeloma. *Lancet Oncol* **17**, e328-e346 (2016). https://doi.org/10.1016/S1470-2045(16)30206-6

9 Walker, B. A. *et al.* Mutational Spectrum, Copy Number Changes, and Outcome: Results of a Sequencing Study of Patients With Newly Diagnosed Myeloma. *J Clin Oncol* **33**, 3911-3920 (2015). https://doi.org/10.1200/JCO.2014.59.1503

10 Bolli, N. *et al.* Heterogeneity of genomic evolution and mutational profiles in multiple myeloma. *Nat Commun* **5**, 2997 (2014). https://doi.org/10.1038/ncomms3997
