# Supplementary material for: Deciphering response dynamics and treatment resistance from circulating tumor DNA after CAR T-cells in multiple myeloma
Source: Nat Commun. 2025 Feb 20;16:1824. doi: 10.1038/s41467-025-56486-6 (PMC11842827; doi:10.1038/s41467-025-56486-6)
Supplement: Supplementary file 3 — Reporting Summary [file 41467_2025_56486_MOESM3_ESM.pdf]

Corresponding author(s): David Kurtz
Last updated by author(s): Nov 23, 2024

Reporting Summary

Nature Portfolio wishes to improve the reproducibility of the work that we publish. This form provides structure for consistency and transparency in reporting. For further information on Nature Portfolio policies, see our Editorial Policies and the Editorial Policy Checklist.

Statistics

For all statistical analyses, confirm that the following items are present in the figure legend, table legend, main text, or Methods section.

n/a	Confirmed	
☐	☒	The exact sample size (*n*) for each experimental group/condition, given as a discrete number and unit of measurement
☐	☒	A statement on whether measurements were taken from distinct samples or whether the same sample was measured repeatedly
☐	☒	The statistical test(s) used AND whether they are one- or two-sided<br>*Only common tests should be described solely by name; describe more complex techniques in the Methods section.*
☐	☒	A description of all covariates tested
☐	☒	A description of any assumptions or corrections, such as tests of normality and adjustment for multiple comparisons
☐	☒	A full description of the statistical parameters including central tendency (e.g. means) or other basic estimates (e.g. regression coefficient) AND variation (e.g. standard deviation) or associated estimates of uncertainty (e.g. confidence intervals)
☐	☒	For null hypothesis testing, the test statistic (e.g. *F*, *t*, *r*) with confidence intervals, effect sizes, degrees of freedom and *P* value noted<br>*Give P values as exact values whenever suitable.*
☐	☒	For Bayesian analysis, information on the choice of priors and Markov chain Monte Carlo settings
☐	☒	For hierarchical and complex designs, identification of the appropriate level for tests and full reporting of outcomes
☐	☒	Estimates of effect sizes (e.g. Cohen's *d*, Pearson's *r*), indicating how they were calculated

Our web collection on statistics for biologists contains articles on many of the points above.

Software and code

Policy information about availability of computer code

Data collection	Whole genome sequencing data was obtained from two sources. Data for diffuse large B-cell lymphoma were downloaded from the International Cancer Genome Consortium data portal on May 7, 2018. Data for multiple myeloma were downloaded from dbGAP (accession phs000345.v2.p1) on Feb 12, 2021. For samples collected at our center, sequencing libraries were prepared as described in the methods and sequenced on the Illumina HiSeq or NovaSeq platform using 2x150bp paired end reads. Primary processing of sequencing data was performed with BWA (0.5.9-r16), samtools (versions 0.1.18 and 1.3.1), and bedtools (2.15.0).
Data analysis	Statistical analyses were performed using MATLAB (R2023a), R (version 2023.06.1), and or with GraphPad Prism (version 10.0.3). The contribution of known mutational processes to isolated SNVs from sequencing data was assessed with the deconstructSigs R package using the COSMIC signature set (v3.3) as described in the methods. Copy numbers were assessed by calculating z-score from on-target and off-target sequencing reads.

For manuscripts utilizing custom algorithms or software that are central to the research but not yet described in published literature, software must be made available to editors and reviewers. We strongly encourage code deposition in a community repository (e.g. GitHub). See the Nature Portfolio guidelines for submitting code & software for further information.

Data

Policy information about availability of data

All manuscripts must include a data availability statement. This statement should provide the following information, where applicable:

- Accession codes, unique identifiers, or web links for publicly available datasets
- A description of any restrictions on data availability
- For clinical datasets or third party data, please ensure that the statement adheres to our policy

The genes included in the CAPP-Seq panel, anonymized and summarized clinical and demographic data, and detailed case-level somatic genetic features (i.e. SNVs) from patients where CAPP-Seq was used for disease detection are provided.

Research involving human participants, their data, or biological material

Policy information about studies with human participants or human data. See also policy information about sex, gender (identity/presentation), and sexual orientation and race, ethnicity and racism.

Reporting on sex and gender	This study is a correlative study on patients with plasma cell disorders treated as part of standard of care. Study enrollment was offered regardless of sex or gender. We collected data on sex from medical record. Our cohort consists of 64 patients with 24 female patients, which the proportion is similar to newly diagnosed myeloma patients in general population.
Reporting on race, ethnicity, or other socially relevant groupings	We did not use socially relevant categorization variables in this manuscript.
Population characteristics	In this manuscript, patients with diagnosis of plasma cell disorders were enrolled, either at the time of diagnosis or at relapse. They underwent conventional or cellular therapies under standard of care. Patients were categorized by staging (R-ISS), presence of high-risk cytogenetics of cancer cells, presence of extramedullary disease, and presence of oligo/non-secretory disease.
Recruitment	Patients were recruited at Divisions of Hematology or Blood and Marrow Transplant & Cellular Therapy at Stanford Cancer Center. This is a single-center study, thus may not reflect general population.
Ethics oversight	This study was approved by Stanford Institutional Review Board.

Note that full information on the approval of the study protocol must also be provided in the manuscript.

Field-specific reporting

Please select the one below that is the best fit for your research. If you are not sure, read the appropriate sections before making your selection.

[x] Life sciences [ ] Behavioural & social sciences [ ] Ecological, evolutionary & environmental sciences

For a reference copy of the document with all sections, see nature.com/documents/nr-reporting-summary-flat.pdf

Life sciences study design

All studies must disclose on these points even when the disclosure is negative.

Sample size	The goal of our study is to demonstrate the feasibility of detecting circulating tumor DNA (ctDNA) in multiple myeloma. Using our previous data on lung cancer, where ctDNA has been used in clinical practice, we hypothesized that mean allele frequency of ctDNA in myeloma is 3% higher (1% vs 4%) with population variance of 25%. A sample size of 44 is needed to detect this difference with 80% power and alpha=0.05. This justifies our sample size of 64 with 6 patients with precursor conditions (MGUS/smoldering myeloma) and 58 patients with active myeloma.<br>For CAR-T cohort, we aimed to stratify patients based on day 28 ctDNA levels. We powered our study to detect a meaningful difference (55% difference in progression-free survival at 3 months) between ctDNA negative and positive patients on day 28. A sample size of 20 patients is needed to detect this difference with 80% power and alpha=0.05 (assuming a 1:1 ratio between detectable and undetectable patients by ctDNA). However, previous literature suggests that ~40% of patients will have undetectable minimal residual disease after CAR-T cells (ide-cel), therefore ~22 subjects are required to achieve the necessary sample size. This justifies our sample size of CAR-T cohort with 36 patients.
Data exclusions	Samples deemed to be technical failures during DNA isolation or library preparation were excluded.
Replication	Demonstration of recurrently detected SNVs was performed in 64 independent patient samples with consistent results. Results describing clinical significance of ctDNA detection was replicated with 16 samples on day 90 after CAR T- cell therapy.
Randomization	Patients and controls were enrolled in the study based on a confirmed diagnosis of multiple myeloma receiving standard of care. Therefore, randomization of individuals to different groups is not applicable.
Blinding	No blinding was performed.

Reporting for specific materials, systems and methods

We require information from authors about some types of materials, experimental systems and methods used in many studies. Here, indicate whether each material, system or method listed is relevant to your study. If you are not sure if a list item applies to your research, read the appropriate section before selecting a response.

Materials & experimental systems

n/a	Involved in the study	
☐	☒	Antibodies
☒	☐	Eukaryotic cell lines
☒	☐	Palaeontology and archaeology
☒	☐	Animals and other organisms
☐	☒	Clinical data
☒	☐	Dual use research of concern
☒	☐	Plants

Methods

n/a	Involved in the study	
☒	☐	ChIP-seq
☒	☐	Flow cytometry
☒	☐	MRI-based neuroimaging

Antibodies

Antibodies used	anti-CD138 microbeads, human (Miltenyi Biotec, catalog no. 130-111-744)
Validation	This anti-CD138 microbeads were validated by staining cells and performing flow cytometry per the manufacturer (https://www.miltenyibiotec.com/US-en/products/macsprep-multiple-myeloma-cd138-microbeads-human.html). Dilution used was 1:5.

Clinical data

Policy information about clinical studies

All manuscripts should comply with the ICMJEguidelines for publication of clinical research and a completedCONSORT checklist must be included with all submissions.

Clinical trial registration	This is not an interventional trial.
Study protocol	N/A
Data collection	Patients were recruited from Feb 2017 to Mar 2023, data was collected retrospectively with a cutoff date of Mar 21th, 2024.
Outcomes	Primary outcomes were time to progression (TTP), which is defined as time from CAR T-cell infusion until disease progression. Our secondary outcomes were disease response defined by International Myeloma Working Group (IMWG) criteria.

Plants

Seed stocks	We did not use plants in this study.
Novel plant genotypes	N/A
Authentication	N/A
